# Human leukocyte antigen evolutionary divergence as a novel risk factor for donor selection in acute lymphoblastic leukemia patients undergoing haploidentical hematopoietic stem cell transplantation

**DOI:** 10.3389/fimmu.2024.1440911

**Published:** 2024-08-19

**Authors:** Xing-Yu Cao, Hai-Fei Zhou, Xiang-Jun Liu, Xiao-Bo Li

**Affiliations:** ^1^ Department of Bone Marrow Transplant, Hebei Yanda Lu Daopei Hospital, Langfang, China; ^2^ Department of Bone Marrow Transplant, Beijing Lu Daopei Hospital, Beijing, China; ^3^ Beijing BFR Gene Diagnostics Co., Ltd, Beijing, China

**Keywords:** human leukocyte antigen (HLA) evolutionary divergence (HED), acute lymphoblastic leukemia, haploidentical hematopoietic stem cell transplantation, donor selection, risk factor

## Abstract

**Introduction:**

The human leukocyte antigen (HLA) evolutionary divergence (HED) reflects immunopeptidome diversity and has been shown to predict the response of tumors to immunotherapy. Its impact on allogeneic hematopoietic stem cell transplantation (HSCT) is controversial in different studies.

**Methods:**

In this study, we retrospectively analyzed the clinical impact of class I and II HED in 225 acute lymphoblastic leukemia patients undergoing HSCT from related haploidentical donors. The HED for recipient, donor, and donor-recipient pair was calculated based on Grantham distance, which accounts for variations in the composition, polarity, and volume of each amino acid within the peptide-binding groove of two HLA alleles. The median value of HED scores was used as a cut-off to stratify patients with high or low HED.

**Results:**

The class I HED for recipient (R_HED^class I^) showed the strongest association with cumulative incidence of relapse (12.2 vs. 25.0%, P = 0.00814) but not with acute graft-versus-host disease. The patients with high class II HED for donor-recipient (D/R_HED^class II^) showed a significantly higher cumulative incidence of severe aGVHD than those with low D/R_HED^class II^ (24.0% vs. 6.1%, P = 0.0027). Multivariate analysis indicated that a high D/R_HED^class II^ was an independent risk factor for the development of severe aGVHD (P = 0.007), and a high R_HED^class I^ had a more than two-fold reduced risk of relapse (P = 0.028). However, there was no discernible difference in overall survival (OS) or disease-free survival (DFS) for patients with high or low HED, which was inconsistent with the previous investigation.

**Discussion:**

While the observation are limited by the presented single center retrospective cohort, the results show that HED has poor prognostic value in OS or DFS, as well as the associations with relapse and aGVHD. In haploidentical setting, class II HED for donor-recipient pair (D/R_HED^class II^) is an independent and novel risk factor for finding the best haploidentical donor, which could potentially influence clinical practice if verified in larger cohorts.

## 1 Introduction

Allogeneic hematopoietic stem cell transplantation (allo-HSCT) is a curative therapy for many hematopoietic disorders, including acute lymphoblastic leukemia (ALL) (1). The success of allo-HSCT partly depends on the recognition of tumor antigens presented to alloreactive T cells via human leukocyte antigens (HLAs). The importance of HLA matching is currently well established, resulting in a fully HLA-matched sibling or unrelated donor being the preferred source for allo-HSCT to reduce the risk of GVHD through allo-recognition of foreign HLA molecules (2, 3).

The divergence of HLA alleles may lead to an increased functional capability of the immunopeptidome, which would defend against potentially fatal opportunistic infections and leukemia cells causing relapse (4). Heterozygosity was typically used to assess the HLA allelic difference. Recently, HLA evolutionary divergence (HED), a metric reflecting the immunopeptidome diversity, has been utilized to more accurately quantify HLA allele divergence using the Grantham distance, which accounts for variations in the composition, polarity, and volume of each amino acid within the peptide-binding groove of two HLA alleles (5, 6). Previous research has linked the high heterozygosity of HLA class I loci to an improved response to immune checkpoint inhibitors in advanced cancer patients (7). Further, Chowell et al. found that the effect of HED on survival was independent of other clinically relevant variables and that a high HED in class I alleles was strongly related with response to checkpoint inhibitors in advanced cancer patients (8). These findings, however, were subsequently challenged by a study with a large cohort of cancer patients who had undergone anti-PD1 immunotherapy (9).

In the context of liver grafts, Feray et al. discovered that the donor’s HED was an intrinsic feature completely independent of the recipient’s characteristics and that a high class I HED of the donor was strongly related to a poor outcome (10). The influence of class I and II HED in the HSCT setting has primarily been explored in acute myeloid leukemia (AML). In AML patients, a high class I/class II HED ratio was revealed to be an independent factor for improved overall and disease-free survival (11, 12). More recently, HED was utilized to predict the outcome of children and young adults who underwent HSCT from an unrelated donor for a variety of malignant disorders (4). According to this study, patients with a high HED score of the combined HLA-B and -DRB1 loci had significantly increased overall and disease-free survival.

As an alternative donor transplant, HLA-haploidentical transplantation allows patients who do not have fully matched donors to undergo a transplant, and it has been increasingly used globally over the last two decades (13). In the haploidentical HSCT setting, almost all patients have more than one donor. As a result, the search for the best donor is a critical issue because donor selection can considerably affect the incidences of graft-versus-host, relapse, transplant-related mortality, and survival (13). Previous studies have identified a variety of characteristics that influence haploidentical outcomes, including HLA matching, donor age, donor sex, family relationships, and so on. These risk factors should be considered when selecting the best donor. However, the effects of HLA disparity on transplantation outcomes have vanished due to the improved protocols of haploidentical HSCT with anti-thymocyte globulin (ATG) or with post-transplantation cyclophosphamide (PT/Cy). If HLA disparity, either the quantity of HLA-mismatched loci or the mismatch combination of specific sites, is not a risk factor for haploidentical donor selection, it is currently unclear whether HED, which reflects HLA allele spatial epitope information, affects donor selection and clinical outcomes (4). To date, little is known about the impact of HED on outcomes in the HLA-haploidentical HSCT setting. In this study, we scored HED for donors, recipients, and donor-recipient pairs, and assessed the clinical significance of class I and II HED in 225 ALL patients who received HLA-haploidentical HSCT from a related donor. We found that the Grantham distance score of HLA evolutionary divergence was associated with acute GVHD and relapse in ALL patients undergoing HLA-haploidentical HSCT from a related donor, which may be considered a novel risk factor for donor selection in the haploidentical transplant setting.

## 2 Materials and methods

### 2.1 Patient characteristics

To investigate the influence of HED on clinical outcomes following HSCT, we conducted a retrospective analysis of consecutive Acute Lymphoblastic Leukemia patients (ALL) receiving allo-HSCT between 2012 and 2017 at Hebei Yanda Lu Daopei Hospital, Langfang City, PR China. HED was calculated using data from all patients. The clinical data collected included graft-versus-host disease (GVHD), relapse, date of the event, survival status, and last follow-up date, etc. All patients were prepared for transplantation using modified myeloablative or reduced intensity conditioning regimens (based on total body irradiation, busulfan, or fludarabine, depending on the patient’s comorbidities) (14). According to Chinese Bone Marrow Transplant Cooperative Group recommendations, GVHD prophylaxis was based on anti-thymoglobulin (ATG), cyclosporin A (CsA), methotrexate (MTX), and mycophenolate mofetil (MMF) (15–17).

This retrospective study was reviewed and approved by the Ethics Committee of Hebei Yanda Lu Daopei Hospital (DEPC-M-2023, No. 20). Before data collection, written informed consent was obtained from the patient or the patient’s parents if the patient was under the age of 18. This study follows the Declaration of Helsinki.

### 2.2 HED calculation

HLA compatibility was determined at five loci (HLA-A, -B, -C, -DRB1, and -DQB1) using sequencing-based typing (SBT) GenDx excellerator kits (GenDX, Utrecht, Netherlands). The patient and donor two-field resolution typing of these HLA loci served as the input for the HED calculation, and the calculation was performed using a Python script according to the original Grantham distance formula presented in the literature (5).

**Figure 1:**
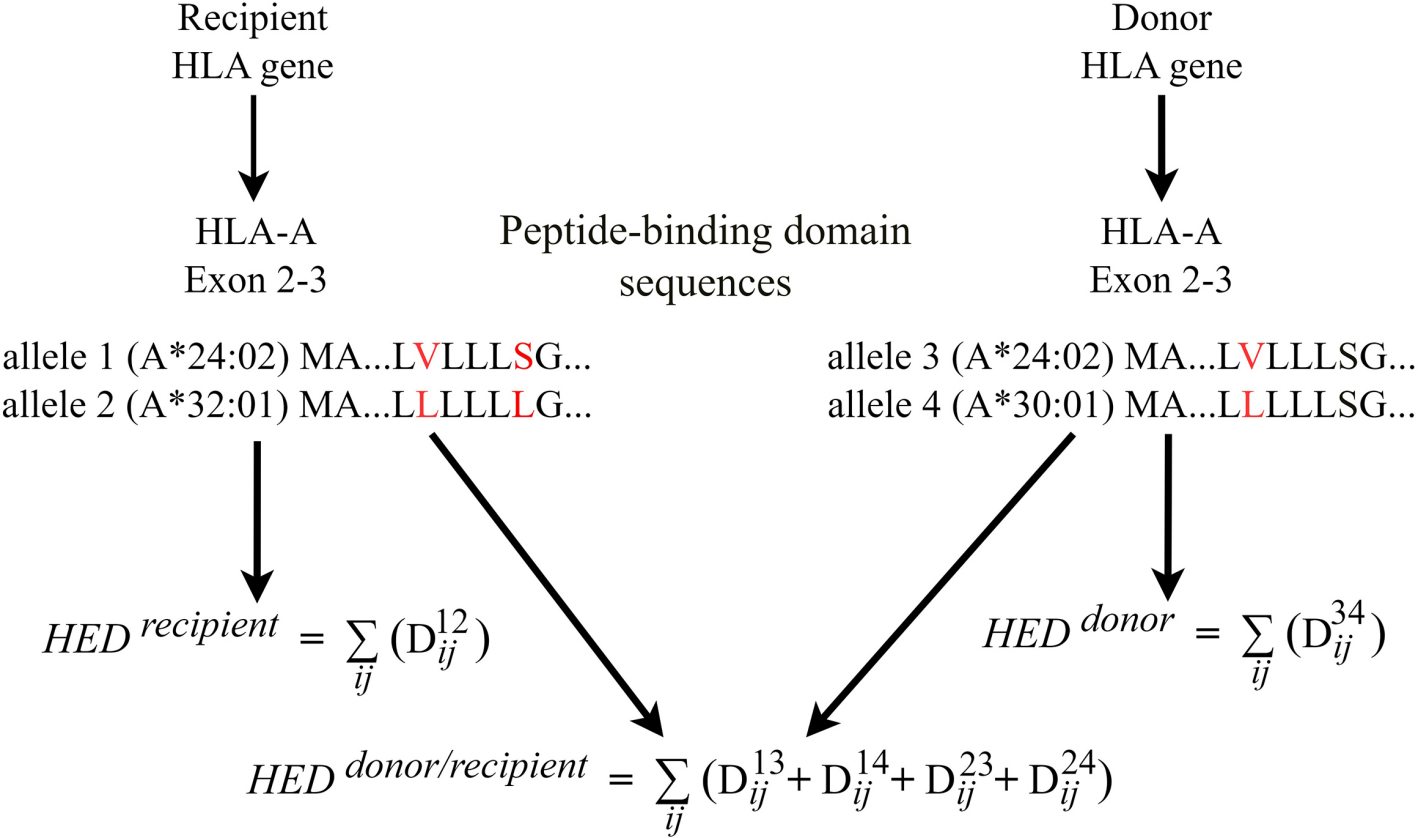
HED calculation flowchart for donors, recipients and donor-recipient pairs.


For each donor and recipient, the HED score was determined by calculating the Grantham distance between the peptide-binding domains of the two alleles at the HLA loci (exons 2 and 3 for HLA-A, HLA-B and HLA-C, exons 2 for HLA-DQB1, HLA-DRB1) loci (6, 7). For donor-recipient pair, HED per locus was estimated for pairwise allele combinations between donors and recipients. We take HLA-A as an example to illustrate how HED between donors and recipients was calculated (**Figure 1**). If recipient has HLA-A allele 1 and 2, donor has HLA-A allele 3 and 4 (**Figure 1**). *D_ij_* is Grantham distance between two alleles and calculated using the original formula (5) as follows:

$$D_{ij}={\left[\alpha {\left(c_{i}-c_{j}\right)}^{2}+\beta {\left(p_{i}-p_{j}\right)}^{2}+\gamma {\left(v_{i}-v_{j}\right)}^{2}\right]}^{1/2}$$

Where *i* and *j* represent paired amino acids of the same position in the sequence of two alleles. *c*, *p* and *v* represent respective composition, polarity and molecular volume of the homologous amino-acids at a given position. *α*, *β* and *γ* are constants. HED between donor and recipient (*HED ^donor/recipient^*) was calculated by the sum of Grantham distance of four combinations for donor-recipient alleles, given by the formula:


$$HED^{donor/recipient}=\sum_{ij}\left({\text{D}}_{ij}^{13}+{\text{D}}_{ij}^{14}+{\text{D}}_{ij}^{23}+{\text{D}}_{ij}^{24}\right)$$

In the context of haploidentical HSCT, where donor and recipient always have one allele shared in any HLA locus, as shown in the diagram allele 1 = allele 3, the formula is:


$$HED^{donor/recipient}=\sum_{ij}\left({\text{D}}_{ij}^{14}+{\text{D}}_{ij}^{23}+{\text{D}}_{ij}^{24}\right)=HED^{donor}+HED^{recipient}+\sum_{ij}\left({\text{D}}_{ij}^{24}\right)$$

Furthermore, if HLA-A matched (allele 1 = allele 3, allele 2 = allele 4), the formula is:


$$HED^{donor/recipient}=HED^{donor}+HED^{recipient}$$

The mean HED score of class I HLA (HED^class I^) or class II HLA (HED^class II^) was measured for donor, recipient, and donor-recipient, respectively. HED was denoted by the prefix R (Recipient), D (Donor), or D/R (Donor-Recipient pair). The median HED score was used as the threshold to define a high- or low-HED group.

### 2.3 Clinical endpoints

The primary objective was to assess the impact of HED on relapse, non-relapse mortality (NRM), and acute and chronic graft-versus-host disease (GVHD). The secondary aim of the study was to assess the effect of HED on prognosis following haploidentical HSCT.

Endpoints of interest included the cumulative incidence of GVHD, relapse and NRM, overall survival (OS), and disease-free survival (DFS). aGVHD incidence was defined as time to first diagnosis of aGVHD (grade 2-4). Because acute GVHD, especially of grade 2 or higher, is probably the most suitable marker of morbidity, an additional sub-analysis for aGVHD (grades 3-4) was performed. Patients who survived more than 14 and 100 days following transplantation were evaluated for acute and chronic GVHD, respectively. The modified Keystone Criteria were used to grade aGVHD (18), while the National Institute of Health Consensus Criteria were used to evaluate cGVHD (19). Relapse incidence was defined as the time to relapse and death without prior recurrence. The NRM event was treated as a competing risk for relapse. NRM was defined as the time to death from any cause other than relapse. OS was defined as the time from transplantation to death, or the last follow-up. DFS was defined as the probability of survival without disease at any period following transplantation, with relapse or death considered events.

At the last follow-up, patients free from the event of interest were censored. The presence of 5% or more leukemic cells in the bone marrow and no indication of extramedullary localization was considered a hematological relapse.

### 2.4 Statistical analysis

Patient characteristics were summarized using descriptive statistics. Categorical variables are reported as counts (%), while continuous variables are described as the medians. The chi-square test, or Fisher’s exact test, was used to assess differences in categorical variables across two groups. The Mann-Whitney U test was used to compare the intergroup continuous variables.

Cumulative incidences of GVHD, relapse, and NRM were estimated with the methods of Fine and Gray considering the respective competitive risks; comparisons between the high and low HED groups were performed with Gray’s test. The Kaplan-Meier survival curve was used to estimate the probability of OS and DFS, and the significance was determined with a log-rank test. Potential risk factors were identified using the univariate Cox regression method to assess the hazard ratio (HR) for the various factors associated with clinical outcomes. Multivariate Cox regression analysis retained significant HED and other variables that might have been clinically meaningful or statistically significant in univariate analysis (P<0.2). The final multivariate models were built using a backward stepwise model approach.

Variables considered in the multivariate models were donor sex, donor and patient age, donor-recipient HLA disparity, donor-recipient family relationship, disease status at transplant (non-remission vs. complete remission), and donor-recipient sex matching. KIR matching and the HSCT-specific comorbidity index were not included due to insufficient data.

All tests were two-sided, and *P<*0.05 was considered statistically significant. The date collected is as of December 31, 2017. Statistical analysis was performed using the SPSS 25 package (SPSS Inc., Chicago, USA) and a graphical user interface for R language, EZR version 1.32 (20).

## 3 Results

### 3.1 Patient characteristics

The study comprised 225 ALL patients who had HSCT from a related donor between 2012 and 2017. Most of the transplants (179) were parents as donors. Thirty-nine transplants were siblings as donors. The median age was 15 years, with the range of 2 to 48 years, and the median follow-up time following transplantation was 35.8 months (range, 1-83.9). High-resolution HLA typing revealed that 146 (64.9%) of 225 donor-recipient pairs had five mismatches, 43 (19.1%) had four mismatches, and 36 (16.0%) had three or fewer HLA mismatches. Thirty-one individuals (13.8%) had active disease at the time of transplantation. **Table 1** summarizes the patient demographics and characteristics.

**Table 1:** Patient characteristics.

Variable		N	% or range
Age at transplant (yr.)	Median	15	2-48
Gender	Female	81	36%
	Male	144	64%
Donor-recipient relationship	Parent-child	179	79.6%
	Child-parent	7	3.1%
	Sibling-sibling	39	17.3%
HLA-matching	5/10	146	64.9%
	6/10	43	19.1%
	7/10	23	10.2%
	8/10	8	3.6%
	9/10	5	2.2%
Disease status at HSCT	CR1	103	45.8%
	CR2	73	32.4%
	Active disease	49	21.8%
Conditioning regimen	MAC	101	44.9%
	RIC	124	55.1%
TBI	Yes	213	94.7%
	No	12	5.3%
Acute GVHD	Yes	123	54.7%
Chronic GVHD	Yes	161	71.6%
Time from HSCT to aGVHD occurrence (days)	Median	60	4-240
Time from HSCT to cGVHD occurrence (days)	Median	180.5	28-4170
Time from HSCT to relapse (days)	Median	984.5	18-2491
CMV reactivation		144	64.0%
EBV infection		73	32.4%

CMV, cytomegalovirus; CR, complete remission; EBV, Epstein-Barr virus; GVHD, graft-versus-host disease; MAC, myeloablative conditioning; HLA, human leukocyte antigen; HSCT, hematopoietic stem cell transplantation; RIC, reduced intensity conditioning; TBI, total body irradiation.

### 3.2 HED scores

We estimated HED strictly following the original formula of Grantham distance. Our HED value for class I was 3.56 times higher than Pierini and Lenz’s (6), and for class II, it was 1.75 times higher (Supplementary Method). This discrepancy is due to differences in data processing, but there is a clear and straightforward relationship between the two calculation methods, thus they can be considered identical in clinical investigations.

**Figure 2:**
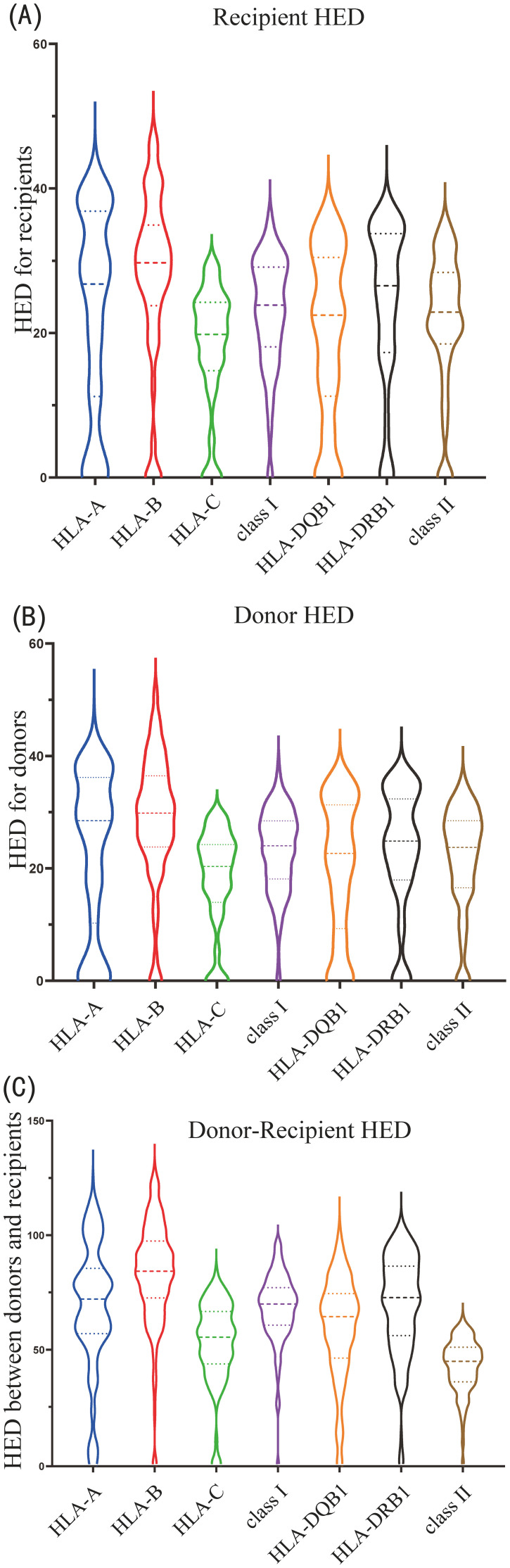
Violin plot of HLA evolutionary divergence **(HED)** distribution for **(A)** recipients, **(B)** donors, and **(C)** donor-recipient pairs.

For recipients, HLA-B locus showed the highest HED variability (R_HED^B^, median 29.7), followed by HLA-A (R_HED^A^, median 26.8), HLA-DRB1 and -DQB1 (R_HED^DRB1^ and R_HED^DQB1^, median 26.5 and 22.5, respectively), and HLA-C locus displayed the lowest HED variation (R_HED^C^, median 19.8) (**Figure 2A**). HLA-B evolutionary divergences were greater than HLA-A and HLA-C, supporting previous findings that HLA-B is the most ancient and diverse of the three HLA-class I loci (6). Class I HLA had a slightly higher mean HED (R_HED^class I^) than class II HLA (R_HED^class II^) (median 23.9, 22.9, respectively) (**Figure 2A**). The variance and distribution pattern of donor HED were quite comparable to that of the recipient, with HLA-B having the highest value (D_HED^B^, median 29.8) and HLA-C having the lowest (D_HED^C^, median 20.3) (**Figure 2B**).

Despite the fact that the HED scores for donor-recipient pairs were much higher than those of the donor or recipient due to the computed divergence among the four alleles, the HED distribution and variation patterns of each locus or class were identical to those of the donor or recipient. The highest was D/R_HED^B^ (median 84.2), followed by D/R_HED^DRB1^ (median 72.7), D/R_HED^A^ (median 72.0), and D/R_HED^DQB1^ (median 64.4), while the lowest was D/R_HED^C^ (median 55.4). D/R_HED^class I^ was higher than D/R_HED^class II^ (median 69.9 versus 67.4) (**Figure 2C**).

### 3.3 GVHD

**Figure 3:**
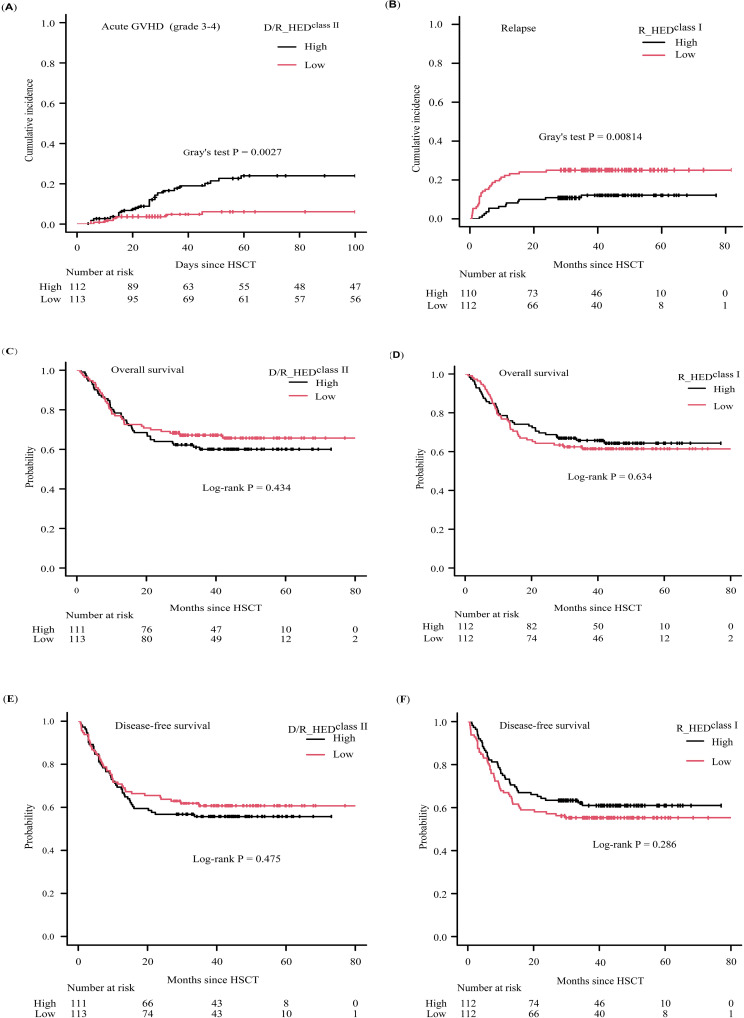
Clinical outcomes according to D/R_HED^class II^ and R_HED^class I^ **(A)** cumulative incidence of aGVHD (grade3-4) stratified by D/R_HED^class II^, **(B)** cumulative incidence of relapse stratified by R_HED^class I^, **(C)** KM curve of overall survival stratified by D/R_HED^class II^, **(D)** KM curve of overall survival stratified by R_HED^class I^, **(E)** KM curve of disease-free survival stratified by D/R_HED^class II^, **(F)** KM curve of disease-free survival stratified by R_HED^class I^.

The overall cumulative incidences of grade 2-4 and 3-4 aGVHD at 100-day were 36.5% (95% confidence interval [CI]: 29.9-43.0%), and 15.1% (95% CI: 10.3-20.7%), respectively. Neither the donor (D_HED^class I^, D_HED^class II^) nor recipient HED values (R_HED^class I^, R_HED^class II^) had any effect on aGVHD. Surprisingly, the HED score of donor-recipient pair (D/R_HED^class II^) was significantly associated with the cumulative incidence of grade 3-4 aGVHD at 100-day. The incidence of grade 3-4 aGVHD was 24.0% (95%CI:15.7-33.3%) in patients with high D/R_HED^class II^ compared to 6.1% (95%CI: 2.5-12.2%) in patients with low D/R_HED^class II^ (P = 0.0027) (**Table 2**, **Figure 3A**). The favorable impact of D/R_HED^class II^ appears to be primarily driven by D/R_HED^DRB1^. The higher the D/R_HED^DRB1^, the higher the incidence of grade 3-4 aGVHD (23.4% [95%CI:15.1-32.8%] vs 7.2% [95%CI: 3.1-13.5%], P = 0.0047).

**Table 2:** Cumulative incidences (%) of Relapse, NRM, cGVHD and aGVHD based on HED^class I^ and HED^class II^.

Factor	Group	Relapse*	NRM*	aGVHD^§^	cGVHD*
D_HED^class I^	High	11.8 (6.6-18.6)	21.9 (14.7-30.1)	16.6 (9.7-25.2)	79.4 (69.4-86.5)
	Low	25.2 (17.5-33.7)	23.2 (15.9-31.4)	13.5 (7.5-21.3)	78.9 (68.4-86.3)
	P value	0.0123	0.808	0.928	0.666
R_HED^class I^	High	12.2 (6.8-19.3)	25.5 (17.7-33.9)	14.7 (8.4-22.7)	82.7 (72.0-89.6)
	Low	25.0 (17.4-33.3)	19.7 (12.9-27.6)	15.4 (8.8-23.7)	76.7 (66.4-84.2)
	P value	0.00814	0.246	0.858	0.355
D/R_HED^class I^	High	12.7 (7.3-19.7)	23.6 (16.2-31.9)	18.3 (11.1-26.8)	78.8 (68.6-86.0)
	Low	24.2 (16.7-32.5)	21.5 (14.4-29.5)	11.6 (6.0-19.1)	80.1 (69.6-87.2)
	P value	0.0232	0.631	0.331	0.533
D_HED^class II^	High	18.1(11.5-25.9)	24.2 (16.7-32.5)	18.1 (10.8-26.9)	79.6 (69.3-86.7)
	Low	19.1(12.3-27.0)	20.9 (13.8-29.0)	12.1 (6.5-19.5)	79.0 (68.6-86.3)
	P value	0.796	0.556	0.402	0.702
R_HED^class II^	High	15.8 (9.6-23.4)	20.2 (13.2-28.2)	15.3 (8.8-23.6)	74.8 (64.2-82.6)
	Low	21.2 (14.2-29.2)	24.9 (17.3-33.2)	14.7 (8.4-22.8)	84.3 (74.2-90.7)
	P value	0.293	0.421	0.903	0.0886
D/R_HED^class II^	High	19.3 (12.5-27.2)	23.9 (16.3-32.2)	24.0 (15.7-33.3)	73.0 (62.0-81.3)
	Low	18.0 (11.4-25.7)	21.3 (14.3-29.4)	6.1(2.5-12.2)	85.6 (75.8-91.7)
	P value	0.775	0.674	0.0027	0.0311

*Cumulative incidence (%) at 5 years; §Incidence of grade 3-4 aGVHD at 100 days; NRM, non-relapse mortality; Numbers in parenthesis indicate 95% Confidence Interval. HED’s prefix D, R, and D/R indicate donor, recipient and donor-recipient pair, respectively.

The 5-year cumulative incidence of cGVHD was unexpectedly high, at 80.4% (95%CI: 74.1-86.0%). In contrast to the results for aGVHD, the cumulative incidence of cGVHD at 5-year was significantly associated with higher D/R_HED^class II^ (P = 0.0311), with higher D/R_HED^class II^ being associated with lower cGVHD risk (73.0% vs. 85.6%), but not with D/R_HED^class I^ (P = 0.533) (**Table 2**). D/R_HED^class II^ was therefore included in the subsequent cox regression analysis for GVHD. Regardless of the negative association with D/R_HED^B^, there was no significant correlation between cGVHD and D/R_HED^class I^.

### 3.4 Relapse and NRM

Forty-four of 225 (19.6%) patients relapsed at a median time of 984.5 days (range 18-2491) after transplantation. The 5-year cumulative incidence of relapse (CIR) for all patients after transplantation was 18.6% (95% CI: 13.7-24.0%). The cumulative incidence of NRM at five years was 22.6% (95% CI: 17.3-28.3%), which was greater than the 5-year CIR.

When patients are stratified based on HED^class I^ or HED^class II^, all three HED^class I^ (D_HED^class I^, R_HED^class I^, and D/R_HED^class I^) scores show an obvious association with CIR (**Table 2**). Higher D_HED^class I^ and D/R_HED^class I^ contribute to a lower 5-year CIR (11.8 vs. 25.2%, P = 0.0123; 12.7% vs. 24.2%, P = 0.0232) (**Table 2**). R_HED^class I^, in particular, exhibited the strongest association with 5-year CIR (12.2 vs. 25.0%, P = 0.00814) (**Table 2**, **Figure 3B**). Conversely, neither HED^class II^ were correlated with 5-year CIR. Thus, the three HED^class I^(D_HED^class I^, R_HED^class I^, and D/R_HED^class I^) were used as candidate risk factors for subsequent Cox regression analysis. The cumulative incidence of NRM at five years was not associated with any HED^class I^ or HED^class II^. These findings suggest that genetic divergence of class I HLA, rather than class II HLA, may be responsible for the differences in CIR, but that genetic differentiation of either class I or II HLA loci has little effect on NRM.

### 3.5 Multivariate analysis

The impact of HED on GVHD and relapse was further investigated using the Cox proportional hazard regression analysis with consideration of other risk factors in multivariate analysis. The univariate analysis for GVHD, relapse and DFS is shown in **Supplementary Table 1**.

The multivariate regression analysis revealed that the low R_HED^class I^ group had a more than two-fold greater risk of relapse (HR = 2.101 [95%CI: 1.083-4.078], P = 0.028) (**Table 3**, **Figure 3B**, **Supplementary Table 2**), whereas non-remission patients exhibited an approximately threefold risk of relapse. Therefore, R_HED^class I^ can be considered an independent risk factor for relapse.

**Table 3:** Significant factors for GVHD and relapse in multivariate analyses.

Outcomes	HR	95% CI	P value
Relapse^*^			
R_HED^class I^, low vs high	2.101	1.083-4.078	0.028
Disease status, NR vs CR	2.928	1.440-5.951	0.003
aGVHD (grade 3-4) ^*^			
D/R_HEDclass II, low vs high	0.335	0.148-0.756	0.009
cGVHD			
Model 1^*^			
D/R_HED^class II^, low vs high	1.376	0.995-1.904	0.054
Model 2^§^			
Donor age, >=45 vs <45	1.738	1.160-2.603	0.007
aGVHD status	1.438	1.024-2.018	0.036

^*^Donor age was treated as continuous variable. ^§^Donor age was treated as dichotomous variable. CR, complete remission; NR, non-remission; aGVHD and cGVHD, acute and chronic graft-versus-host disease.

In the multivariate model of severe aGVHD, the low D/R_HED^class II^ significantly reduced the risk of grade 3-4 aGVHD (HR = 0.335 [95% CI: 0.148-0.756], P = 0.009) as the only protective factor when considering donor age as a continuous variable (**Table 3**; **Supplementary Table 2**). However, when donor age was considered a dichotomous variable, it remained in the final model as a risk factor but failed to reach a statistically significant level (HR = 2.153, P = 0.068) (**Table 3**; **Supplementary Table 2**).

Regarding cGVHD, Model 1 with donor age as a continuous variable revealed that D/R_HED^class II^ was the only independent risk factor, and low D/R_HED^class II^ was associated with high risk of cGVHD (HR = 1.376 [95% CI: 0.995-1.904]); however, this association reached marginal statistical significance (P = 0.054, **Table 3**; **Supplementary Table 2**). In Model 2, patients with a history of aGVHD or receiving transplantation from donor older than 45 years had a high risk for cGVHD development, while D/R_HED^class II^ no longer remained significant.

### 3.6 Survival

The proportions of 5-year OS and DFS for the entire cohort were 62.9% (95% CI: 56.1-68.9%) and 58.2% (95% CI: 51.4-64.4%), respectively. There was no discernible difference in overall and disease-free survival (OS or DFS) between patients with high- and low-HED (**Figures 3C–F**; **Supplementary Figures 1**, **2**) except D_HED^class I^ associated with DFS, implying that HED was ineffective as a prognostic indicator for ALL patients who underwent HLA-haploidentical HSCT with related donors. Multivariate regression analysis confirmed that HED, including D_HED^class I^, was not associated with survival.

## 4 Discussion

In the present investigation, we report the impact of HED scores on clinical outcomes for ALL patients underwent haploidentical HSCT. This represents the first study investigating HED in a pure cohort of haploidentical transplantations with patients affected by only one type of hematologic malignancy. While the observation are limited by the presented single center retrospective cohort, the results find that HED has poor prognostic value in OS/DFS, as well as the associations with relapse and aGVHD. In haploidentical setting, HLA disparity was once considered to have little impact on transplantation benefits, but our results showed that HED is an independent risk factor for selecting the best haploidentical donor.

Previous studies investigated the impact of HED on prognosis in mixed AML patients transplanted from either related or unrelated HLA-matched donors (11, 12). Roerden et al. examined the effect of HED on survival in an AML cohort with an HLA-identical sibling or foreign donor and found that a high class I HED had a favorable impact on OS (12). In AML patients undergoing HSCT, Daul et al. investigated the effect of class I and II HED on survival using four different donor sources: identical siblings, haploidentical donors, matched unrelated donors, and mismatched unrelated donors (11). The authors claimed that the class I/II HED ratio was an independent factor associated with better DFS/OS and could be an additive indication of GVL in addition to the major allogenic effect associated with the mismatched HLA. Recently, various hematological diseases were examined in a study by Merli et al., which supports the use of HED as a predictive marker in young adult and pediatric patients receiving transplantation from unrelated donors (4).

The ability of immune cells to interact with mismatched HLAs, minor histocompatibility antigens, and tumor-associated antigens (TAAs) on the leukemic cells is the foundation of the GVL effect (graft-versus-leukemia) (21). Compared to related patient/donor pairs, the overall genetic divergence for unrelated patient/donor pairs is higher. According to whole exome sequencing of patient-donor pairs undergoing allo-HSCT, an average of 6,445 non-synonymous SNVs were found to be mismatched, offering a sizable pool of possible miHAs (22). Genome-wide SNP array analyses revealed that the average mismatched SNVs in the coding region were 9.4% for sibling donors, rising to 17.3% for unrelated donors (23). To lessen the confounding effect of genetic background divergence, we therefore restricted the analysis to a pure cohort of haploidentical transplantation recipients who received transplantation from the related donor.

Our data showed that high R_HED^class I^ was associated with a lower 5-year CIR, confirming the crucial function of CD8^+^ effective T cells in the GVL immune response and thus directly reflecting the immunological benefit of high HED. Patients with high HED scores potentially exhibit more immunogenic peptides than those with low HED scores, which may be recognized by donor-derived T lymphocytes (24), thereby reducing the likelihood of relapse. This explanation can be supported by similar research conducted recently. It was found that AML patients with high class I HED tended to recover their CD8^+^ T, B, and NK cells more quickly (11). Recently, Pagliuca et al. found that high recipient class I HED was associated with a higher diversity of TCR repertoire (25). In the first year of HSCT, a higher diversity of TCR repertoire and enhanced immune reconstitution might result in a strong defense against opportunistic infections (4).

However, our studies did not reveal any differences in OS or DFS between high and low R_HED^class I^ALL patients, indicating that high R_HED^class I^ was not always associated with a good prognosis as seen in AML. Patients with high R_HED^class I^ had a relatively high incidence of NRM (25.5%) despite a low relapse rate (12.2%) (**Table 2**), which in turn offset the survival benefit from high R_HED^class I^, resulting in no significant difference in OS. This possible explanation is related to the Beijing protocol we used. The difference between our results and those of earlier studies may also be due to differences in disease type and ethnicity. Our cohort enrolled ALL patients, which has characteristics that cannot be totally extrapolated from studies of AML patients. For instance, while AML is incredibly sensitive to NK cell alloreactivity, the majority of adult ALL patients are not (26, 27). Furthermore, our homogeneous cohort is limited to Chinese, and distinct HLA alleles and HLA haplotypes are present in each ethnic group (28), emphasizing the significance of studying HED in this particular population. It’s interesting to note that Chhibber et al. (2022) found that genetic diversity of class I or II HLA loci (HED, heterozygosity, genotype) was not associated with clinical outcomes (9), suggesting that this biomarker shouldn’t be used for clinical decision-making for cancer patients receiving pembrolizumab. Similar studies conducted independently have also confirmed Chhibber’s conclusion (29–31). To properly comprehend the overall impact of HED, therefore, more research in larger cohorts and across more centers would be required.

As an alternative donor transplant, haploidentical HSCT offers patients who lack fully matched donors the chance to receive transplant, while donor-derived alloreactive T cells elicit a strong allogeneic response and exert an immense GVL effect (32). Between 2005 and 2015, there was a roughly threefold increase of haplo-HSCT in Europe due to favorable practical aspects of using a haploidentical donor and the accumulation of data of better outcomes achieved with TCR platforms (33). The democratization of using haploidentical donors leads to a fundamental paradigm shift: while donor availability was the key challenge for years, the issue today becomes identifying the best donor among several possible ones when haplo-HSCT (34). In general, the outcome of haploidentical HSCT may be influenced by DSA (donor-specific antibody), donor age, donor sex, KIR (killer immunoglobulin-like receptor), NIMA (noninherited maternal antigen), HLA matching, as well as family relationships (35, 36). Recent studies have confirmed that neither the quantity of HLA loci nor the combination of specific sites would affect the outcome of haploidentical HSCT (35, 37–40). The Beijing protocol showed that 1, 2, or 3 mismatches of 6 HLA loci had no effect on the cumulative incidence of cGVHD or aGVHD. Additionally, the number of HLA mismatches had no influence on the cumulative incidence of relapse, overall survival, and leukemia-free survival (35, 37). The cumulative incidence of GVHD, relapse rate, NRM, and overall survival were not affected by differences in the HLA locus in the T-cell-replete (TCR) haploidentical HSCT with a low dose of anti-T lymphocyte globulin (ATG), according to a prospective multicenter study from Japan (39). In the multivariate analysis, the only significant predictive factor for increased relapse was non-CR status prior to transplantation (P = 0.0424), which tended to be associated with a worse survival rate (P = 0.0524). It was also observed that the degree of HLA mismatching had no effect on post-transplant OS, cumulative incidence of aGVHD, NRM, or 1-year cGVHD in the high-dose PT/Cy haploidentical transplantation protocol, whether in the HVG (host-versus-graft) or GVH (graft-versus-host) settings (40). According to Kasamon et al., survival following nonmyeloablative transplants with posttransplant cyclophosphamide is also not correlated with the degree of HLA disparity (41).

Technique advances in aGVHD prophylaxis, prevention of post-transplant relapse, and treatment strategies have greatly improved the outcome of haploidentical HSCT compared to the past decades. Although the team from the Beijing protocol established the notion of donor selection and the best option of donor selection is to choose youthful, male, and NIMA-incompatible donors (35, 36), the consensus of donor selection, however, is still limited within the TCD and TCR haploidentical systems at this time. New criteria for donor selection may develop as a result of an increase in haploidentical HSCT cases and updated assessments of the factors influencing transplant outcomes (33, 34, 42–44). In this study, we found a strong association between D/R_HED^class II^ and aGVHD incidence, with higher D/R_HED^class II^ indicating more severe aGVHD. Single locus analysis revealed that the influence of D/R_HED^class II^ appears to be predominantly driven by D/R_HED^DRB1^,which proves the conclusions that DRB1 has the highest diversity among all HLA class II genes and the highest cell surface expression when compared to other HLA class II antigens (45). A high D/R_HED^class II^ implies great spatial structural differences between donors and recipients, as well as more targets from tissue cells being presented. As a result, the greater the effect of T-cells attacking the tissue cells, the more severe the damage to the organ. The number of mismatch loci is obviously a relatively rough indicator, although it also reflects the degree of incompatibility between recipient and donor. Therefore, previous studies and our results suggest that the amount of HLA mismatch should not be used as a criterion for the selection of family haploidentical donors. Instead, D/R_HED^class II^ provides more epitope information than mismatch numbers and also indicates donor and recipient mismatches, suggesting that D/R_HED^class II^ may be taken into account as a new risk factor for donor selection in related haploidentical HSCT.

There are some limitations to this study. Our research was based on single-center and retrospective data and had a limited number of patients. Independent, prospective, larger, and multicenter investigations would be needed and beneficial to further confirm the impact of HED on outcome and the clinical significance of D/R_HED^class II^ in donor selection. Due to the unavailability of data or limitations of the methods themselves, other approaches such as peptide binding motifs (PBM) (46), T-cell epitope (TCE) (47) or KIR-ligand mismatches (34, 45) were not considered.

In conclusion, we conducted a retrospective analysis to investigate the correlation between HED and outcomes in ALL patients who underwent transplants from related haploidentical donors. Results revealed that only class I HED of the recipient (R_HED^class I^) was associated with 5-year CIR and only D/R_HED^class II^ was significantly correlated with severe aGVHD. Multivariate Cox regression analysis did confirm that a high D/R_HED^class II^ was an independent risk factor for grade 3-4 aGVHD, and the high R_HED^class I^ group had a more than two-fold reduced risk of relapse. KM and multivariate regression analyses confirmed that none of HED was associated with overall or disease-free survival. These results suggest that HED^class II^ of donor-recipient pair could be used for donor selection as a novel risk factor for grade 3-4 aGVHD and patient’s HED^class I^ for relapse in the setting of related haploidentical HSCT, but not as an independently prognostic factor for predicting OS or DFS.

## Data Availability

The original contributions presented in the study are included in the article/supplementary material. Further inquiries can be directed to the corresponding author/s.
