# Supplementary figures and images for: Human leukocyte antigen evolutionary divergence as a novel risk factor for donor selection in acute lymphoblastic leukemia patients undergoing haploidentical hematopoietic stem cell transplantation
Source: Front Immunol. 2024 Aug 19;15:1440911. doi: 10.3389/fimmu.2024.1440911 (PMC11369896; doi:10.3389/fimmu.2024.1440911)

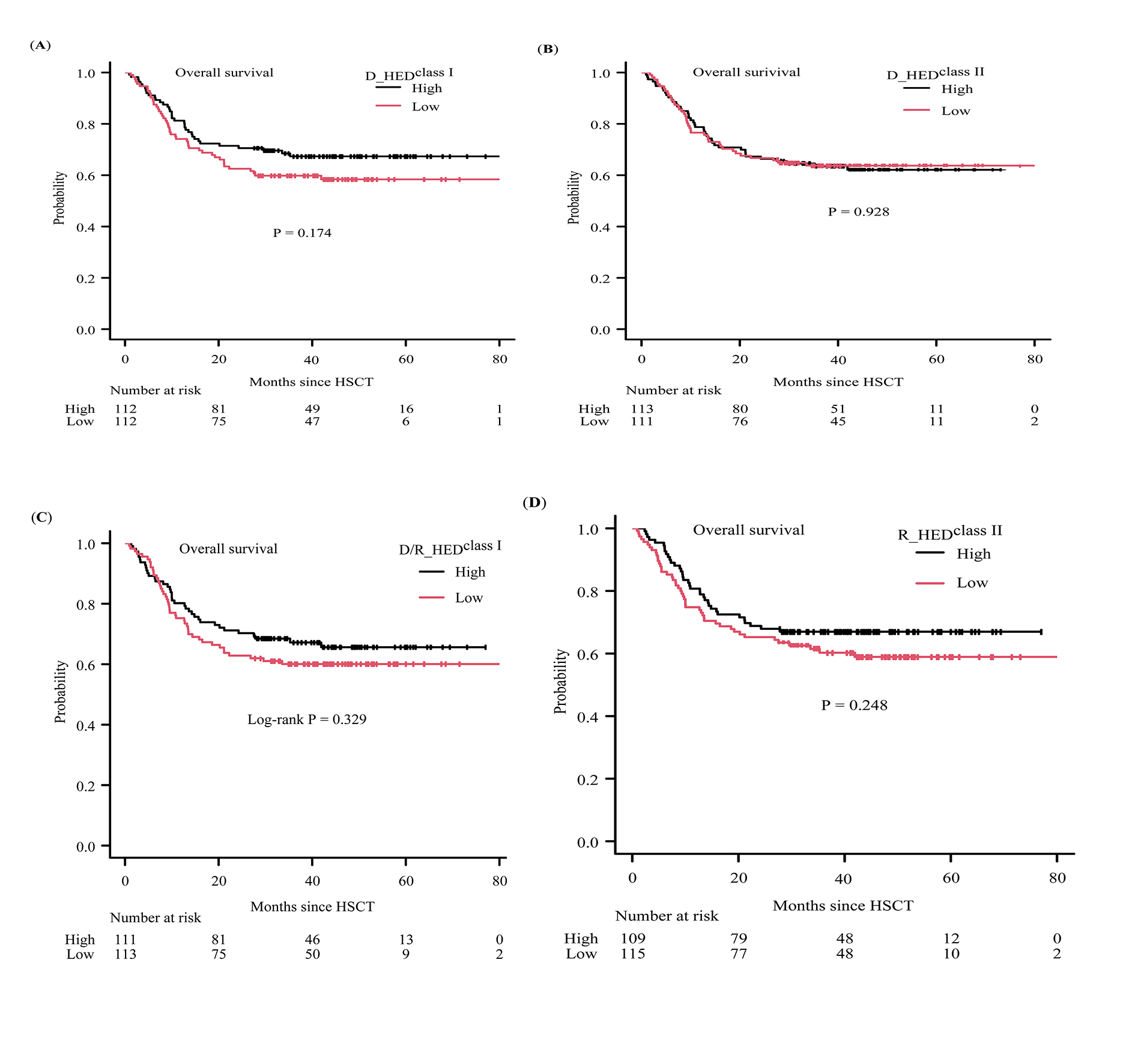

Supplement: Supplementary file 1 [file Image1.jpeg]

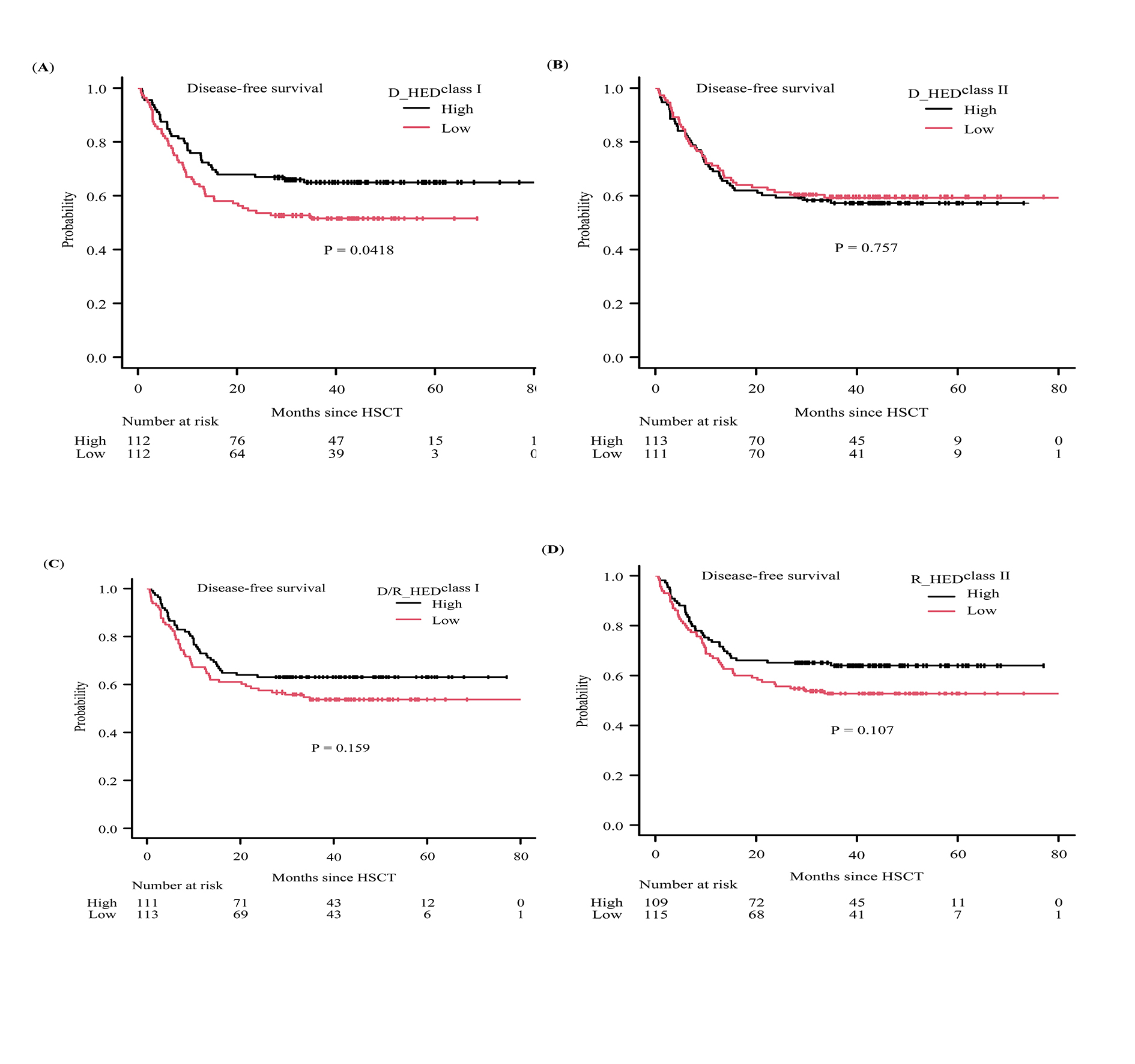

Supplement: Supplementary file 2 [file Image2.jpeg]
