# Supplementary material for: Human leukocyte antigen evolutionary divergence as a novel risk factor for donor selection in acute lymphoblastic leukemia patients undergoing haploidentical hematopoietic stem cell transplantation
Source: Front Immunol. 2024 Aug 19;15:1440911. doi: 10.3389/fimmu.2024.1440911 (PMC11369896; doi:10.3389/fimmu.2024.1440911)
Supplement: Supplementary file 5 [file DataSheet1.pdf]

Supplementary Method

In 1974, Grantham proposed a improved formula for the overall difference between amino acids, combining three properties that correlate best with protein residue substitution frequencies: composition, polarity, and molecular volume.

We compared our results to Pierini and Lenz's (Mol Bio Evol 2018.35: 2145-58; https://sourceforge.net/projects/granthamdist/). Grantham's equation, $D_{ij} = [\alpha (c_i - c_j)^2 + \beta (p_i - p_j)^2 + \gamma (v_i - v_j)^2]^{1/2}$, was used by both us and Pierini & Lenz to calculate the difference (D value) between any two protein residues i and j. Pierini & Lenz, on the other hand, referred to the data processing method from Table 2 in Grantham's original article for their calculations, which means that the mean chemical distance from the three-property formula D_{cpv} is set to 100, with D_{ij} values multiplied by 50.723 to get the mean. Additionally, Pierini & Lenz divided the Grantham distance by the length of the amino acid sequence, resulting 181 for locus of HLA class I (exon2 and exon3) and 89 for locus of HLA class II (exon2). Taken together, our HED value was 3.56 times (181/50.723) higher than Pierini's for HLA class I, and 1.75 times (89/50.723) higher for HLA class II. Both calculation methods are easily convertible. Anyone who is interested can access our script.
